# Impact of tiered restrictions in December 2020 on COVID-19 hospitalisations in England: a synthetic control study

**DOI:** 10.1136/bmjopen-2024-086802

**Published:** 2025-01-04

**Authors:** Xingna Zhang, Daniel Hungerford, Mark Green, Marta García-Fiñana, Iain Buchan, Benjamin Barr

**Affiliations:** 1 Department of Public Health and Policy, University of Liverpool, Liverpool, UK; 2 Department of Health Data Science, University of Liverpool, Liverpool, UK; 3 National Institute for Health and Care Research Health Protection Research Unit in Gastrointestinal Infections, University of Liverpool, Liverpool, UK; 4 Department of Clinical Infection Microbiology and Immunology, University of Liverpool, Liverpool, UK; 5 Department of Geography and Planning, University of Liverpool, Liverpool, UK; 6 Department of Public Health, Policy and Systems, University of Liverpool, Liverpool, UK

**Keywords:** COVID-19, SARS-CoV-2 Infection, Public health, Hospitalisation

## Abstract

**Abstract:**

**Objectives:**

To evaluate the effectiveness of localised Tier 3 restrictions, implemented in England in December 2020, on reducing COVID-19 hospitalisations compared with less stringent Tier 2 measures and the variations by neighbourhood deprivation and the prevalence of Alpha (B.1.1.7) variant, the primary variant of concern then, to measure hospital services’ burden and inequalities across different communities.

**Design:**

Observational study using a synthetic control method, comparing weekly hospitalisation rates in Tier 3 areas to a synthetic control from Tier 2 areas.

**Setting:**

England between 4 October 2020 and 21 February 2021.

**Participants:**

23 million people under Tier 3 restrictions, compared with a synthetic control group derived from 29 million people under Tier 2 restrictions.

**Interventions:**

Tier 3 restrictions in designated areas were implemented from 7 December 2020, imposing stricter limits on gatherings and hospitality than Tier 2, followed by a national lockdown on 6 January 2021.

**Primary and secondary outcome measures:**

Weekly COVID-19-related hospitalisations for neighbourhoods in England over 11 weeks following the interventions.

**Results:**

Implementing Tier 3 restrictions were associated with a 17% average reduction in hospitalisations compared with Tier 2 areas (95% CI 13% to 21%; 8158 (6286 to 9981) in total). The effects were similar across different levels of neighbourhood deprivation and prevalence of the Alpha variant.

**Conclusions:**

Regionally targeted Tier 3 restrictions in England had a moderate but significant effect on reducing hospitalisations. The impact did not exacerbate socioeconomic inequalities during the pandemic. Our findings suggest that regionally targeted restrictions can be effective in managing infectious diseases.

**STRENGTHS AND LIMITATIONS OF THIS STUDY**

- We used a reliable measurement, COVID-19 hospitalisation, which is less affected by levels of testing and case detection than other transmission indicators, to evaluate the effectiveness of interventions.
- We conducted a detailed neighbourhood-level examination of the impact of Tier 3 localised restrictions on hospital admissions to inform community-targeted public health strategies in England.
- We employed a quasi-experimental study design to derive plausible causal inferences, strengthening the evidence base.
- Our assumption of constant infection hospitalisation rate over our study period may mask some temporal variations in hospitalisation rates.
- We were unable to investigate underlying behaviour changes, biological processes or how Tier 3 effects varied by individual or household characteristics.

## Introduction

 Managing COVID-19 hospitalisations, a vital indicator of the burden on healthcare services, was a major challenge during the pandemic.^1 2^ At the beginning of the pandemic, ‘flattening the curve’ in hospitalisations was crucial in England.^3^ Nevertheless, England was one of the most severely affected countries, with some of the globally highest rates of COVID-19 hospitalisations and deaths between late 2020 and early 2021.^4 5^ From December 2020, England started to experience a rapid growth in COVID-19 hospitalisations with a 7-day rolling average increasing three-fold to middle January 2021. To control the spread and ‘Protect the NHS’ (The National Health Service), a localised three-tier system first introduced in October 2020 was reintroduced in December 2020, following a month-long second national lockdown in between.

During the pandemic, such non-pharmaceutical interventions (NPIs) made crucial contribution, particularly to alleviating hospital strains when effective treatments/vaccines were still widely inaccessible.^6 7^ Literature evaluating NPI effects focuses on viral transmission dynamics on national or global scales,^8^,^11^ while much of the available evidence on COVID-19 hospitalisation derives from simulated predictions on observed socioeconomic and demographic data with compartmental models, having made assumptions about clinical progression characteristics and transmission patterns and dynamics.^9 12 13^ Findings from these studies were pivotal in policymaking to combat the pandemic. However, neighbourhood-level interactions between hospitalisation, clinical progression and transmission following localised NPIs could differ from national or global simulation assumptions.

We did an English literature search on quantifying localised NPI effects on COVID-19 health burdens and found ten relevant studies. One study predicted that physical distancing would have significantly reduced infections in Wuhan (China) by implementing staggered return-to-work in early April 2020.^14^ One study simulated a local (county-by-county) and a global (province-by-province) strategy of reopening/reclosing schools and workplaces in Ontario (Canada) and found the former more effective in controlling COVID-19 infections.^15^ One study in Portugal found that a reduction in the growth rate was observed in very high tier after the implementation of a tiered restriction system.^16^ Two studies in Italy found that transmission substantially decreased in the town of Vo after the community lockdown between 24 February and 8 March 2020^17^ and that tiered restrictions on human activities reduced transmissibility and thus resulted in averting about 36% of the hospitalisations between 6 and 25 November 2020.^18^ Another two studies examined effects of localised restrictions in Chile during 2020: localised lockdowns between 21 March and 4 May reduced new cases in higher-income but not lower-income areas;^19^ localised lockdowns between 3 March and 15 June reduced transmission through strong modulation of the duration and indirect effects of neighbourhood interconnectedness.^20^ Two other studies found tiered restrictions effective in reducing transmissions, using reproduction number between 1 July and 4 November 2020 in the UK^21^ and case number between September 2020 and January 2021 in England.^22^ Only one study examined hospitalisations under all tiers introduced in October 2020 in England and predicted a moderate reduction from then to 31 March 2021.^23^

All ten studies have focused on either predicting future hospitalisation trends or effects on infections, which could be affected by prediction assumptions and levels of testing and case detection. The distinction between measures of infection (such as case number) and healthcare demand (hospitalisations) is crucial, with each measure offering essential insights needed for public health responses and resource planning, particularly during pandemics. Compared with measures of infection, the impact of localised NPIs on COVID-19 hospitalisations has been insufficiently studied using observational methods,^24^,^26^ including in England. Evidence shows that COVID-19 hospitalisation is less affected by case detection and a reliable indicator of the demand/burden of healthcare.^9 13 27^ Localised restrictions usually vary across neighbourhoods within countries that are at different phases of the pandemic with varied levels of resources/resilience.^17 20 22^ Gathering empirical evidence about local contexts is vital to delivering suitable health interventions to avoid overwhelming healthcare facilities and staff in places with high burdens.^9 13^ Such evidence is much needed to better gauge localised healthcare demand, particularly when observed hospitalisation data for neighbourhoods is available now in many countries including England.^28 29^

To fill our knowledge gap and lend support for the ongoing UK COVID-19 inquiry, we, therefore, endeavour to analyse the effects of Tier 3 restrictions introduced in December 2020 on COVID-19 hospitalisations, compared with Tier 2 restrictions in this study. While our previous analysis demonstrated that Tier 3 restrictions effectively reduced infection numbers compared with Tier 2,^22^ it remained uncertain if these reductions would also impact more severe outcomes, such as hospitalisations. This paper addresses this question, aiming to inform strategies for managing healthcare demand in future pandemics. We further evaluate whether these intervention effects varied between small areas by level of deprivation and the prevalence of an Alpha variant (B.1.1.7), the primary variant of concern in England during our study period.

## Methods

### Patient and public involvement

Patients and the public were not involved.

### Setting, data and measures

We used Hospital Episode Statistics data provided by NHS Digital on a weekly number of hospital admissions for COVID-19 (International Classification of Diseases 10th edition: code U07.1 for confirmed infections and U07.2 for suspected or probable infections by clinical or epidemiological diagnosis)^30^ in England between 4 October 2020 and 21 February 2021 as our outcome variable. They were aggregated to Middle Layer Super Output Areas (MSOA), a standard geographical unit in England covering around 20 km^2^ and 8000 residents on average.

We used measures of local area characteristics to adjust for potential confounders that could potentially affect vulnerability to hospitalisations, areal variations in healthcare-seeking behaviour arising from socioeconomic conditions, ethnicity, age and health profiles and/or effectiveness of control measures. These included the overall score of the English Indices of Multiple Deprivation (IMD) 2019—a composite indicator of socioeconomic disadvantage;^31 32^ total population and percentage of the over 70 using 2019’s mid-year estimates from the Office for National Statistics (ONS);^32^ proportion of Black, Asian and mixed ethnicity (BAME);^33^ population density from the ONS^34^ and the proportion of people with previous admissions for chronic disease(s) (cardiovascular disease, diabetes, chronic kidney disease or chronic respiratory disease) during 2014–2018 to measure co-morbidities and clinical vulnerability.^32^ To account for pre-intervention local authority (LA) level accessibility differences to SARS-CoV-2 Polymerase Chain Reaction (PCR) testing and the prevalence of B.1.1.7,^35^ we included the number of LA-level PCR tests per capita from the COVID-19 dashboard and the proportion of LA-level positive tests with PCR S-gene test failure (SGTF) from Public Health England.

We then merged this time series of MSOA weekly data, area characteristics and linked LA data, with a dataset of LA-level tiered restrictions compiled by The Open Data Institute.^36^

### Intervention

Tier 3 intervention was implemented on 7 December 2020 (Monday) by many areas, though officially announced on 2 December 2020. We based our analysis on this initial tier allocation, as tiers of most MSOAs remained unchanged except Kent’s MSOAs switched to a new ‘Tier 4’ on 20 December 2020. Analysing the initial tier allocation could offer a more conservative estimate of effect size and avoid biases of selecting places based on subsequent tier transitions, resembling a trial where tier allocation itself is influenced by the effectiveness of restrictions.

We chose concurrent Tier 2 as the ‘control’ to our intervention. Although both tiers prohibited indoor mixing between households, Tier 3 was more restrictive than Tier 2. Meeting with people outside one’s own household in private gardens was not permitted in Tier 3 areas, while such meetings with up to six people were allowed in Tier 2 areas. Pubs and restaurants were closed in Tier 3 areas except for providing takeaway food but remained open in Tier 2 areas if they served ‘substantial meals’ constituting main courses rather than just snacks.

### Analysis

We applied the synthetic control method for microdata developed by Robbins and Davenport.^37 38^ The synthetic control group is constructed using a weighted combination of non-intervention areas. The intervention effect is estimated by comparing the postintervention trend in outcome between the intervention and the synthetic control group.^39^

Accounting for COVID-19’s incubation and clinical progression, we assumed a 2-week lag from the intervention onset (7 December 2020) to observe impacts on hospitalisation (20 December 2020).^40^,^42^ We measured COVID-19 hospitalisation changes in the intervention and the synthetic control group, 12 weeks before and 9 weeks after 20 December 2020 (between 4 October 2020 and 21 February 2021). We used this extended period to understand the extent to which Tier 3 effects (compared with Tier 2) were sustained.

To create the synthetic control group, we derived calibration weights to match Tier 2 and Tier 3 MSOAs over the pre-intervention period based on previously described eight local area characteristics and baseline hospitalisation levels. The calibrated weights met two conditions: the control group’s weighted average for each of the local area characteristics equalled that of the intervention areas and the total number of COVID-19 hospitalisations in the control group matched that of the intervention areas for each pre-intervention week, minimising potential differences in unobserved characteristics associated with COVID-19 hospitalisation rates or Tier 3 allocation.

We then estimated the average treatment effect for the treated (ATT) as the difference in a cumulative number of COVID-19 hospital admissions in the postintervention period in the intervention areas, compared with the (weighted) cumulative number of admissions in the synthetic control group. To estimate the sampling distribution of the intervention effect and calculate permuted p values and 95% CIs, we generated 250 placebo iterations randomly allocating Tier 2 MSOAs to the intervention group, with sufficiently stabilised and converged distribution of outcomes.^38^

Tier 3 restrictions coincided with widespread community testing (piloted in Liverpool), which had a relatively large effect on hospitalisation.^39^ We thus excluded 342 MSOAs from our analysis (200 in Liverpool City Region and 142 with higher than 1 per 100 population mean weekly lateral flow test (LFT) rate during community testing rollout between 6 November 2020 and 2 January 2021: online supplemental file 1). This left 2809 Tier 3 MSOAs as the intervention group, while the synthetic control was constructed from 3481 Tier 2 MSOAs.

We further conducted interaction analyses to investigate whether the intervention effect varied between MSOAs by deprivation (IMD terciles) and the prevalence of B.1.1.7 (median). We reran the weighting algorithm, stratifying by terciles or median of the relevant variables. The ATT was estimated using stratified calibration weights in a weighted Poisson model with a log link function alongside an interaction term between the stratified variable and the intervention indicator.

To test potential Tier 4 impacts, we experimented with removing Kent’s MSOAs from our analysis (online supplemental file 2). To assess the appropriateness of our research design in adjusting the impacts of community testing, we further carried out a sensitivity test by including 342 MSOAs with higher LFT testing rates (online supplemental file 3). To check the robustness of our approach in potential spatial spill-over effects between neighbouring Tier 2 and 3 areas, we reran the main analysis by excluding the Tier 2 MSOA areas with population-weighted centroids located within 20 km of Tier 3 areas (online supplemental file 4).

All analysis was performed using R V.4.3.2 and the Microsynth package.^37^

## Results

### Descriptive analysis

**Figure 1:**
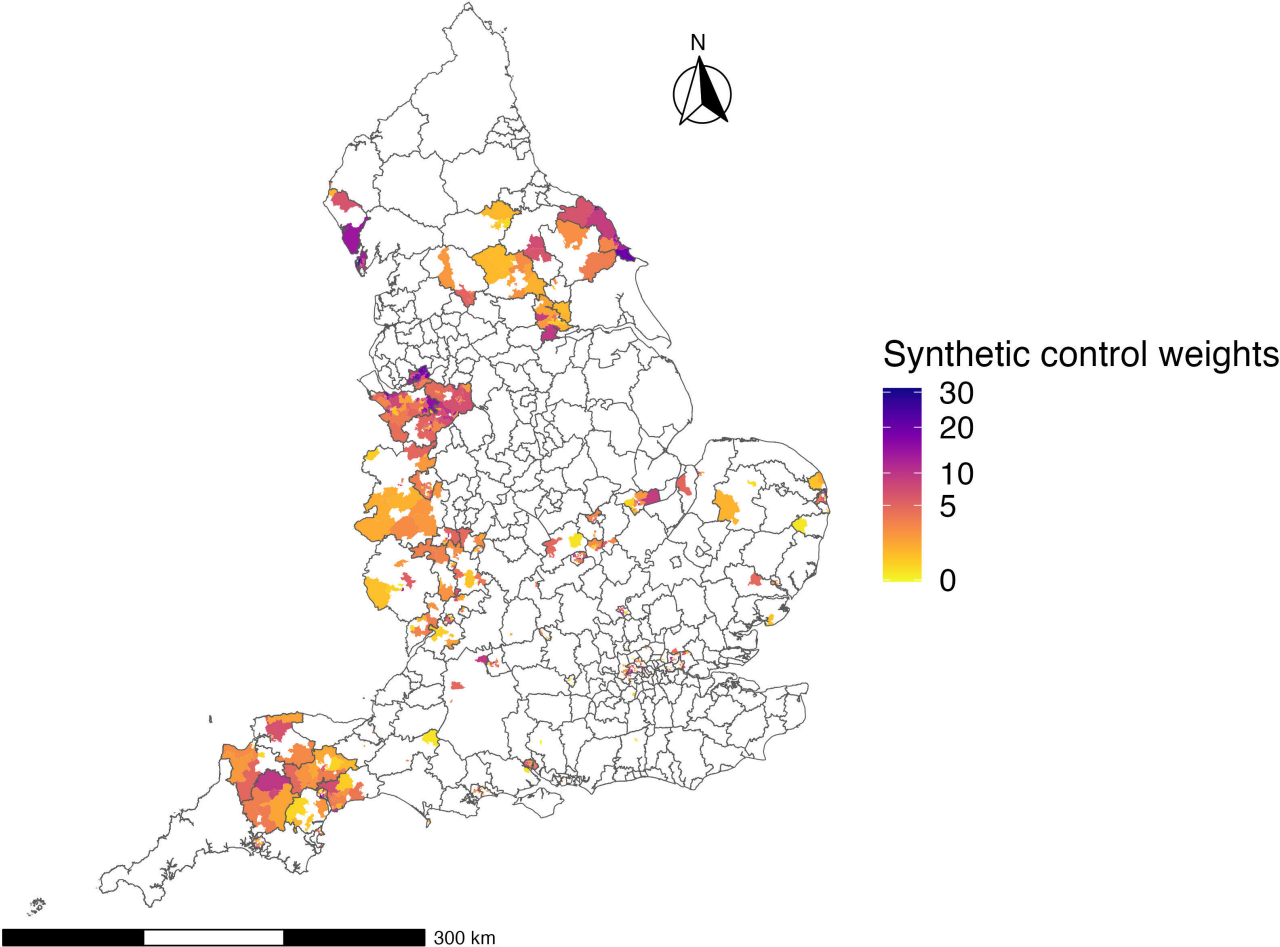
Weighting of Tier 2 Middle Layer Super Output Areas (MSOAs) that were used to construct a synthetic control group at the intervention time point.

We identified 2809 Tier 3 MSOAs as the intervention group and constructed the synthetic control group from the 3481 Tier 2 MSOAs: the brighter the colour, the lower the weights, and vice versa (figure 1). Figure SF4 in online supplemental file 5 shows the location of synthetic control and intervention areas by local authorities.

Table 1 presents summary statistics for the Tier 3 areas and the Tier 2 MSOAs from which the synthetic control group was constructed. Intervention (Tier 3) MSOAs have higher levels of deprivation, lower population density, higher proportion of the population with historical admissions for chronic disease(s), lower proportion of the BAME population, more PCR tests per capita and lower proportion of SGTF. There was no difference in the proportion of the population over 70. In the 12 weeks before the effects of Tier 3 on hospitalisation kicking in on 20 December 2020, Tier 3 areas had a higher COVID-19 admission rate than Tier 2 MSOAs. As the matching algorithm achieved an exact match between the intervention and synthetic control areas, the weighted average of each variable in table 1 would become identical for both groups after applying the calibrated weights.

**Table 1:** Comparison between Tier 3 areas and the MSOAs (Middle Layer Super Output Areas) in the rest of England used to construct the synthetic control group (excluding those within Liverpool City Region or with a high LFT (lateral flow test) testing rate).

	Tier 2 MSOAs used to construct the synthetic control	Tier 3 areas
Index of Multiple Deprivation score	18	26
Population density–people per hectare	42	30
% of population aged 70+ years	14	14
% Black Asian and minority ethnic (BAME)	16	12
% S-gene target failure (SGTF) in routine PCR	43	23
Average Polymerase Chain Reaction (PCR) tests per 100 000 per week in 12 weeks before intervention introduced	2362	2786
% of population with at least one admission for chronic disease(s)	19	21
Average hospital admissions per 100 000 per week for COVID-19 in 12 weeks before intervention introduced	7	14
Total population	29 369 663	22 960 484
Number of MSOAs	3481	2809

### Statistical analysis

**Figure 2:**
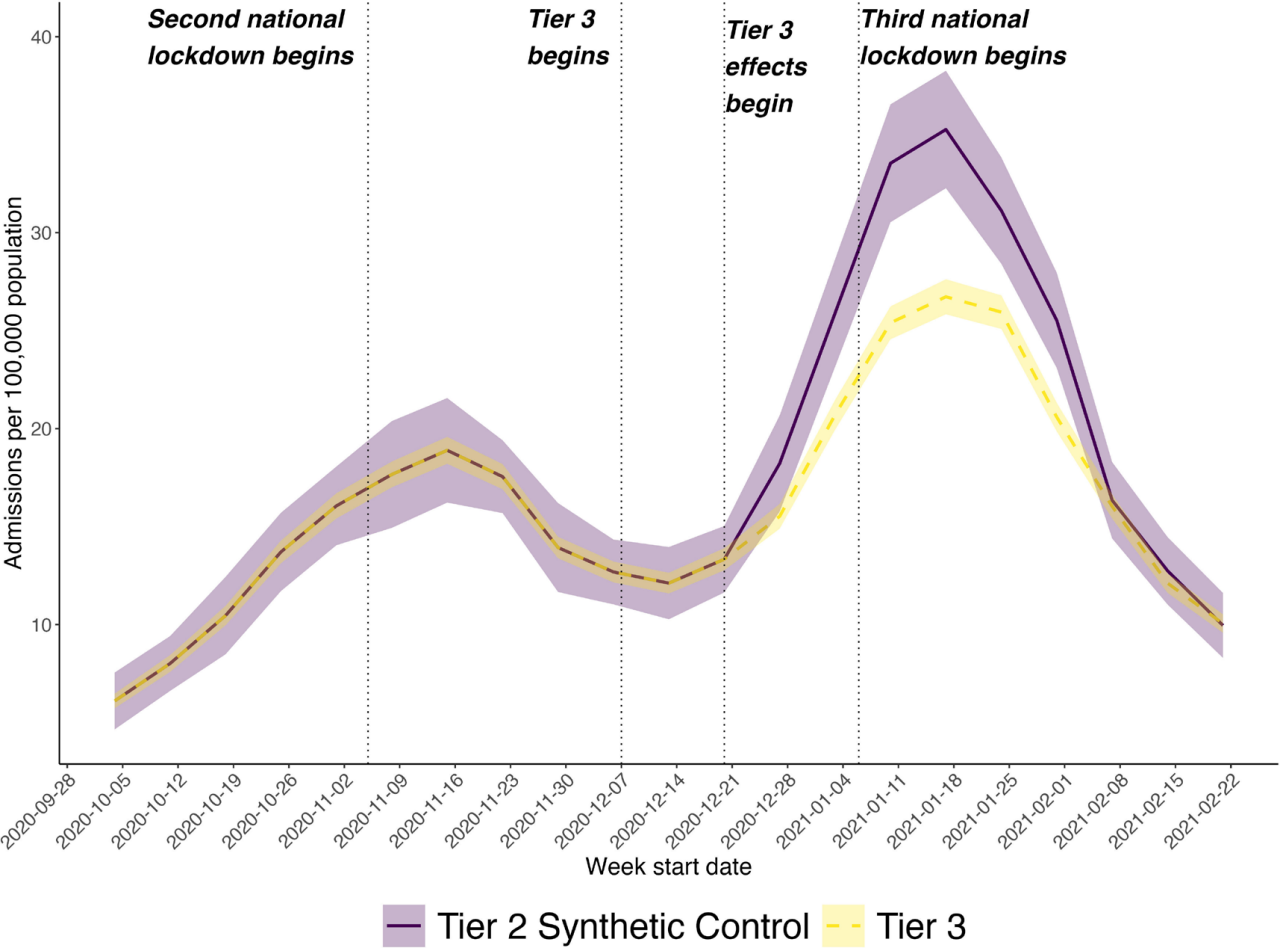
Trend in weekly COVID-19 hospital admission rates in Middle Layer Super Output Areas (MSOAs) in Tier 3 areas compared with a synthetic control group constructed from the weighted average of Tier 2 MSOAs after removing the effect of community testing. Dotted vertical lines represent the start of the second national lockdown on 5 November 2020, followed by the reintroduction of Tier 3 restrictions on 7 December 2020 with a 2-week lag on affecting hospital admissions from 20 December 2020, before the third national lockdown on 6 January 2021.

We visualised the trend in the average weekly COVID-19 hospital admission rates in the intervention and synthetic control areas 12 weeks before and 9 weeks after Tier 3 taking effect on 20 December 2020 (figure 2). These trends were identical in both groups prior to the intervention (4 October to 19 December 2020). In October and the first half of November, the hospitalisation rates were increasing before the effects of the second national lockdown on hospitalisation were felt (assuming the 2-week lag to observe). They then started to fall until mid-December. With the reintroduction of Tier 3 restrictions along with their effects on hospitalisation starting 2 weeks later in December 2020, hospitalisation rates were increasing again until mid-January 2021. This increase however was slower in the Tier 3 areas compared with the synthetic control. From 18 January until 21 February 2021, hospitalisation rates were rapidly falling likely because of the third national lockdown on 6 January 2021. The lower trend in Tier 3 areas continued from late December 2020 to early February 2021, after which no differences were observed between the two groups.

We then estimated the overall effect of Tier 3 restrictions to what would have been expected if Tier 2 restrictions had been applied in those areas (table 2). Tier 3 restrictions implemented in December were associated with an average change of COVID-19 hospital admissions of −17% (95% CI −21% to −13%; total change −8158, 95% CI −9981 to −6286), compared with the synthetic control.

**Table 2:** Results of the synthetic control analysis with a breakdown per level of deprivation (3 levels) and Alpha variant B.1.1.7 (binary following dichotomisation)—indicating the relative reduction in infections in Tier 3 areas compared with what would have been expected if Tier 2 restrictions had been applied. Although differences in effect are observed across levels of deprivation and prevalence of variant B.1.1.7 (indicated by S-gene target failure (SGTF) where quantitative reverse transcription Polymerase Chain Reaction (qRT-PCR) test is used for COVID-19 diagnosis), the interaction terms between level of deprivation and intervention and between SGTF and intervention were not statistically significant.

	Percentage change in admissions (95% CI)	Total change in admissions (95% CI)	P value	P value for interaction between Tier 3 intervention and levels of deprivation and prevalence of the variant respectively
All Tier 3	−17% (−21% to −13%)	−8158 (−9981 to −6286)	<0.001	
Most affluent areas	−19% (−23% to −14%)	−1767 (−2261 to −1216)	<0.001	
Intermediate deprivation	−16% (−22% to −10%)	−1982 (−2742 to −1234)	<0.001	0.434
Most deprived areas	−18% (−26% to −8%)	−4687 (−6756 to −2043)	<0.001	0.841
Lower SGTF (2%–28%)	−17% (−27% to −7%)	−6326 (−10136 to 2553)	<0.001	
Higher SGTF (29%–85%)	−13% (−18% to −8%)	−1154 (−1599 to −670)	<0.001	0.379

In the subgroup analysis by deprivation, the effect size and direction were similar across all levels of deprivation suggesting that the benefits of regional tiers were observed across all levels of socioeconomic deprivation (table 2). Although the effect was slightly greater in both the most deprived and affluent areas, given overlapping confidence intervals, there is no evidence to suggest that deprivation modifies the effects of regional tiers (and no evidence of widening inequalities).

We find evidence for differences in the estimated effect by the level of the median proportion of B.1.1.7 cases. Tier 3 effects may have been greater in areas where the variant was less prevalent (−17%, 95% CIs −27% to −7%; total change −6326, 95% CI −10136 to 2553) than those of higher prevalence (−13%, 95% CIs −18% to −8%; total change −1154, 95% CI −1599 to −670). However, due to overlapping confident intervals and no statistically significant interaction effect, such observation cannot be robustly inferred.

In sensitivity analysis, we repeated the synthetic control models by removing the 182 MSOAs in Kent and found slightly smaller effects (Table SF1 in online supplemental file 2). We also replicated the analysis without excluding the 342 MSOAs with higher LFT testing rates and found slightly larger effects (Table SF2 in online supplemental file 3). We found almost identical effects in testing the potential spatial spill-over effects (Table SF3 in online supplemental file 4).

## Discussion

We found that Tier 3 restrictions implemented in December 2020 were associated with reductions in COVID-19 hospitalisations in local communities (MSOAs) of England, compared with what would be the case if they had been put into Tier 2 restrictions instead. Tier 3 areas had 17% fewer COVID-19 hospital admissions over the study period with the deviation in trends being observed 2 weeks postimplementation of the policy and quickly diverging from trends in Tier 2 areas. This effect was consistent across all levels of deprivation suggesting that that policy did not result in widening inequalities. The effect of regional tiers was potentially higher in areas where B.1.1.7 was less prevalent suggesting that their benefits may only be restricted to less infectious strains. Our study provides novel empirical evidence for the benefits of localised restrictions in managing COVID-19 hospitalisations.^22^

### Strengths

We add to the evidence that localised restrictions on outdoor meeting and the hospitality sector in Tier 3 (compared with Tier 2) had an important role to play in managing COVID-19 hospitalisations. Combined with our previous finding of regional tiers reducing SARS-Cov-2 transmission,^22^ our study here shows that these effects also extend to consequential COVID-19 hospitalisation.^22^ As Tier 3 restrictions were more restrictive in outdoor meeting and the hospitality sector than Tier 2, we unfortunately cannot tell from our analysis whether either one or a combination of both caused the observed effects. Previous evidence indicates that hospitality settings contribute to transmission,^43 44^ but outdoor proximity has a low risk,^45^ which can lead potential reduction on consequential hospitalisations. No consistent evidence supports differentiated effects by levels of deprivation. While national lockdowns required all people to work from home where possible, they may have had different effects across different socioeconomic groups as more deprived people were less likely to work from home.^46^ Additional restrictions on outdoor meeting and the hospitality sector in Tier 3 may have not had such differential effects by socioeconomic group, as transmission in outdoor settings and within the hospitality sector occurred at a similar level across different socioeconomic groups and thus the additional restrictions in Tier 3 reduced risks of consequential hospitalisations by similar amounts.^22^

### Limitations

Our study has limitations. First, we assumed a constant infection hospitalisation rate over our relatively short study period. It could have, however, varied in England due to COVID-19 variants that were more virulent but unaccounted for during our study. Current evidence does not conclusively associate the then primary variant B.1.1.7 (Alpha) with more severe disease than the pre-Alpha variants suggesting our assumption was reasonable.^47^,^50^ Second, although we can match areas to ensure a good balance of potential confounding factors before Tier 3 causing effects, concurrent policy changes and unmeasured confounders could bias the results. Community testing was one affecting transmission and the consequential hospitalisations then.^24^ Although we have sought to account for it, we made the adjustments assuming that the average effects of community testing on hospitalisation were proportional to the transmission levels in Tier 2 and Tier 3 areas in England, coupled with our earlier assumption of the constant infection hospitalisation rate. Kent went into Tier 4 in late December 2020, while results of the sensitivity test removing Kent from our analysis further confirmed the robustness of our main findings (online supplemental file 2). Third, we were only able to use data on neighbourhoods and thus unable to investigate how Tier 3 effects varied by individual or household characteristics. Fourth, we assumed that behavioural trends in intervention and synthetic control areas would be comparable, given their similar characteristics and pre-intervention hospitalisation trends. However, we could not identify specific behavioural changes driving the observed effects on hospitalisation. Some coincidental behavioural changes unrelated to tiered restrictions might have occurred within intervention areas, potentially contributing to these effects. To account for this, we selected a robust outcome—hospitalisation—and adjusted for socioeconomic and demographic confounders of local areas to mitigate biases of areal variations in healthcare-seeking behaviour arising from socioeconomic conditions, ethnicity, age and health profiles. While the observed effects suggest general adherence to regulations, we could not determine whether Peltzman effects—compensatory risk-taking behaviours triggered by safety interventions—affected any individuals in our intervention areas, potentially counteracting the intended benefits. Finally, we could not fit a mechanistic model of COVID-19’s dynamics within our synthetic control design to fully capture the underlying biological processes of transmission, progression and control, although we have attempted to measure the potential spatial clustering of hospitalisations linked by localised infection chains in the online supplemental file 4.

## Conclusions

With increased access to vaccination and the emergence of less deadly variants, many countries, including the UK, lifted COVID-19 restrictions to help people get ‘back-to-normal’. Globally, however, COVID-19 has created large regional and national differences in related disease burden and healthcare needs. Localised restrictions suited to local contexts may remain needed to contain regional outbreaks and reduce pressures on local health systems. Concerns should be raised about the top-down and one-size-fits-all nationwide approaches, given that more deprived areas tend to be more affected economically, and have lower vaccination coverage, poorer healthcare facilities and higher disease burden. Our analysis indicates that tiered restrictions in outdoor gathering and in the hospitality sector are effective at moderately reducing hospitalisation and could be part of an effective strategy for reducing geographical differences in health burden among neighbourhoods as we exit from the pandemic. As the UK conducts a thorough inquiry into the handling of the pandemic, our study provides valuable insights into the effectiveness of community-level interventions. This evidence can inform more adaptable, context-specific public health policies for future crises. A community-level targeted non-pharmaceutical interventions will likely be key components in preparing for and mitigating the impact of future pandemics, enabling health and social policies that are responsive to local needs and socioeconomic factors.

## supplementary material

10.1136/bmjopen-2024-086802 online supplemental file 1

## Data Availability

Data are available in a public, open access repository. Data may be obtained from a third party and are not publicly available.
