# Supplementary material for: Impact of tiered restrictions in December 2020 on COVID-19 hospitalisations in England: a synthetic control study
Source: BMJ Open. 2025 Jan 4;15(1):e086802. doi: 10.1136/bmjopen-2024-086802 (PMC11749879; doi:10.1136/bmjopen-2024-086802)

**SUPPLEMENT**

**Supplement 1: Community testing rates over time.**

**Figure SF1. Trend of mean weekly SARS-CoV-2 antigen lateral flow tests (LFTs) per 100 population across Local Authorities in England between 4^th^ November 2020 and 21^st^ February 2022. Note: Dotted vertical line identifies onsets of community testing pilot in Liverpool and national rollout.**

**
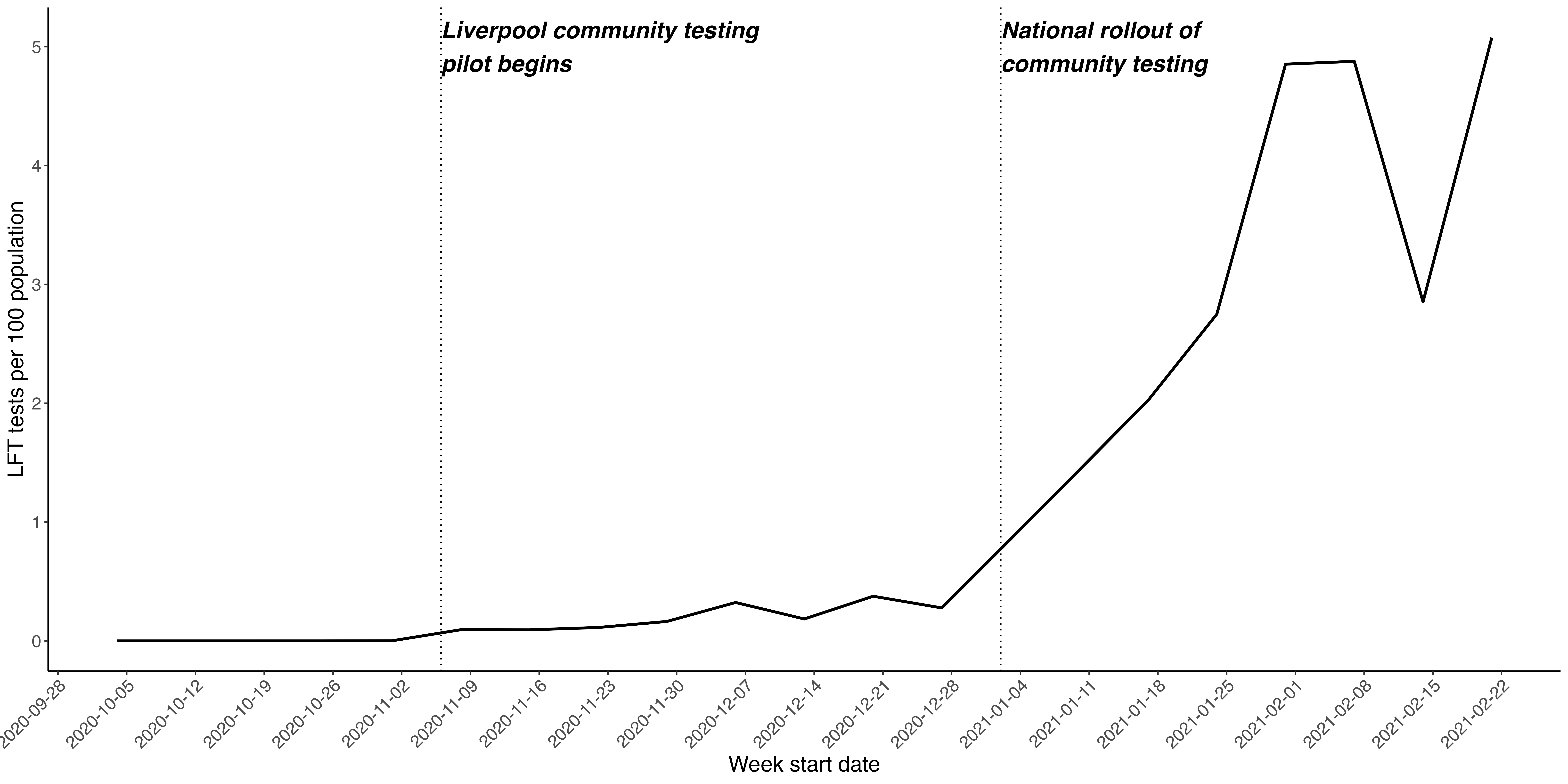
Figure SF2. Distribution of mean weekly SARS-CoV-2 antigen lateral flow tests (LFTs) per 100 population across Local Authorities in England between 6^th^ November 2020 and 2^nd^ January 2021. Note: Dotted vertical line identifies the threshold of the mean LFT testing rate of 1 per 100 population per week that we used to exclude the 142 MSOAs with higher mean LFT rates from our analysis to minimise the potential impact of community testing on hospitalisation.**


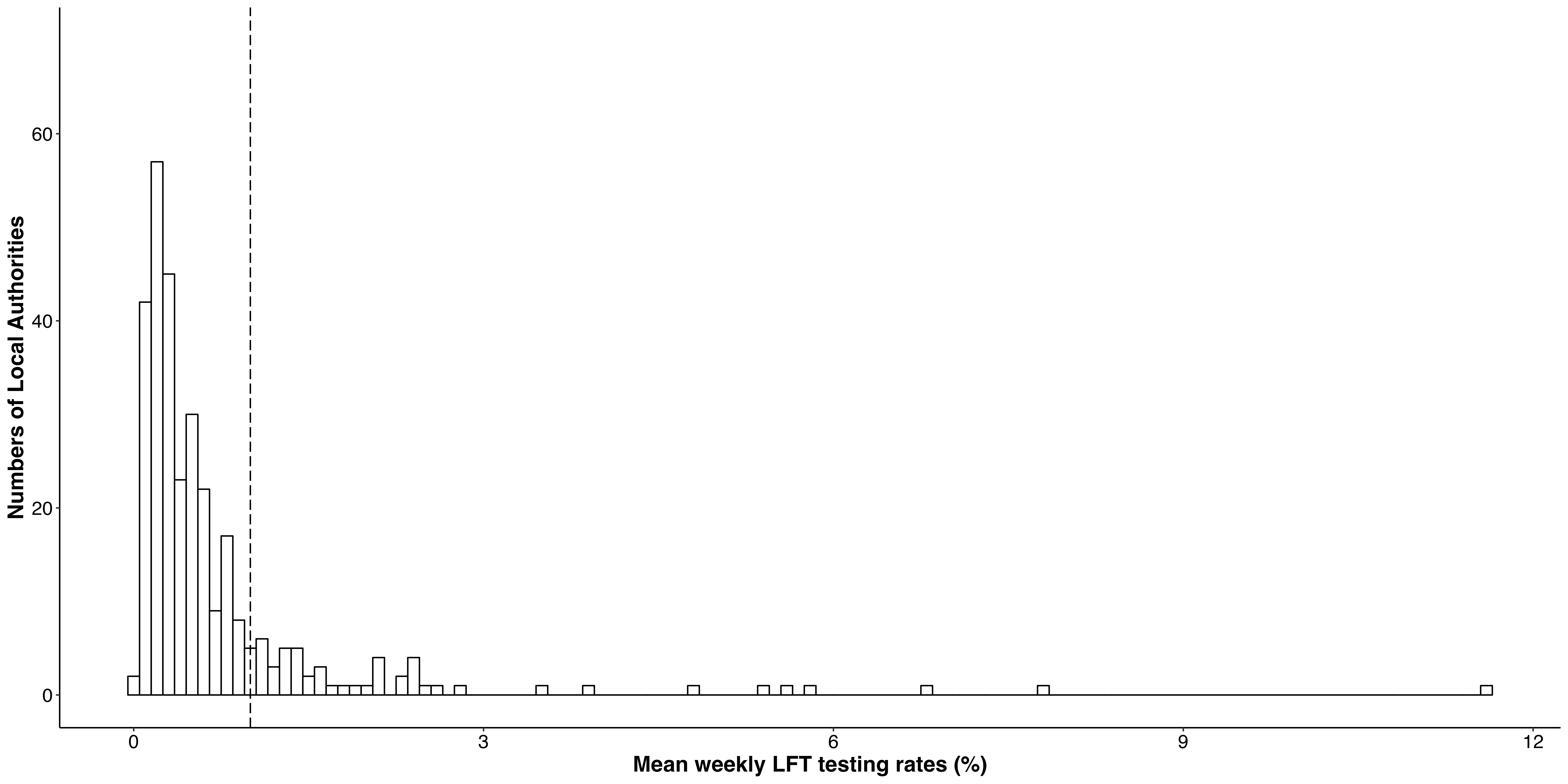


**Supplement 2: Sensitivity test of removing the MSOAs in Kent.**

**Table SF1. Results of the synthetic control analysis after excluding the MSOAs in Kent – indicating the relative reduction in infections in Tier 3 areas compared to what would have been expected if Tier 2 restrictions had been applied.**

|  | Percentage change in cases | 95% CI | | p-value |
| --- | --- | --- | --- | --- |
|  |  | LCL | UCL |  |
| **All Tier 3** | -14% | -20% | -8% | <0.001 |

**Supplement 3: Sensitivity test of including the 200 MSOAs in Liverpool City Region and the 142 MSOAs with mean LFT rates above 1 per 100 population per week.**

**Table SF2. Results of the synthetic control analysis by including the 200 MSOAs in Liverpool City Region and the 142 MSOAs with mean LFT rates above 1 per 100 population per week – indicating the relative reduction in infections in Tier 3 areas compared to what would have been expected if Tier 2 restrictions had been applied.**

|  | Percentage change in cases | 95% CI | | p-value |
| --- | --- | --- | --- | --- |
|  |  | LCL | UCL |  |
| **All Tier 3** | -19% | -23% | -16% | <0.001 |

**Supplement 4: Sensitivity tests of the spatial spill-over effect.**

The distance between MSOA areas is measured by the Euclidean distance of the population weighted centroids of MSOA areas (Data source is ONS Geography Open Data <https://geoportal.statistics.gov.uk/datasets/b0a6d8a3dc5d4718b3fd62c548d60f81_0>). When further excluding the 1,172 Tier 2 MSOA areas located within 20 km of Tier 3 areas (about 34% of the 3,481 eligible MSOAs), we found almost identical result (see Table SF3). This suggests that our results are robust and there probably have been very little spill-over effects. In other words, there is no evidence from our data that traveling from Tier 3 areas to neighbouring Tier 2 areas to take advantage of the less restrictive measures had made any contribution to hospitalisation for COVID-19 in neighbouring Tier 2 areas.

**Table SF3. Results of the synthetic control analysis by excluding the 1,172 Tier 2 MSOA areas located within 20 km of Tier 3 areas – indicating the relative reduction in infections in Tier 3 areas compared to what would have been expected if Tier 2 restrictions had been applied.**

|  | Percentage change in cases | 95% CI | | p-value |
| --- | --- | --- | --- | --- |
|  |  | LCL | UCL |  |
| **All Tier 3** | -16% | -21% | -10% | <0.001 |

**Figure SF3. Location of synthetic control (yellow) and intervention (Tier 3) areas (purple) after excluding the 1172 Tier 2 MSOA areas located within 20 km of Tier 3 areas.**


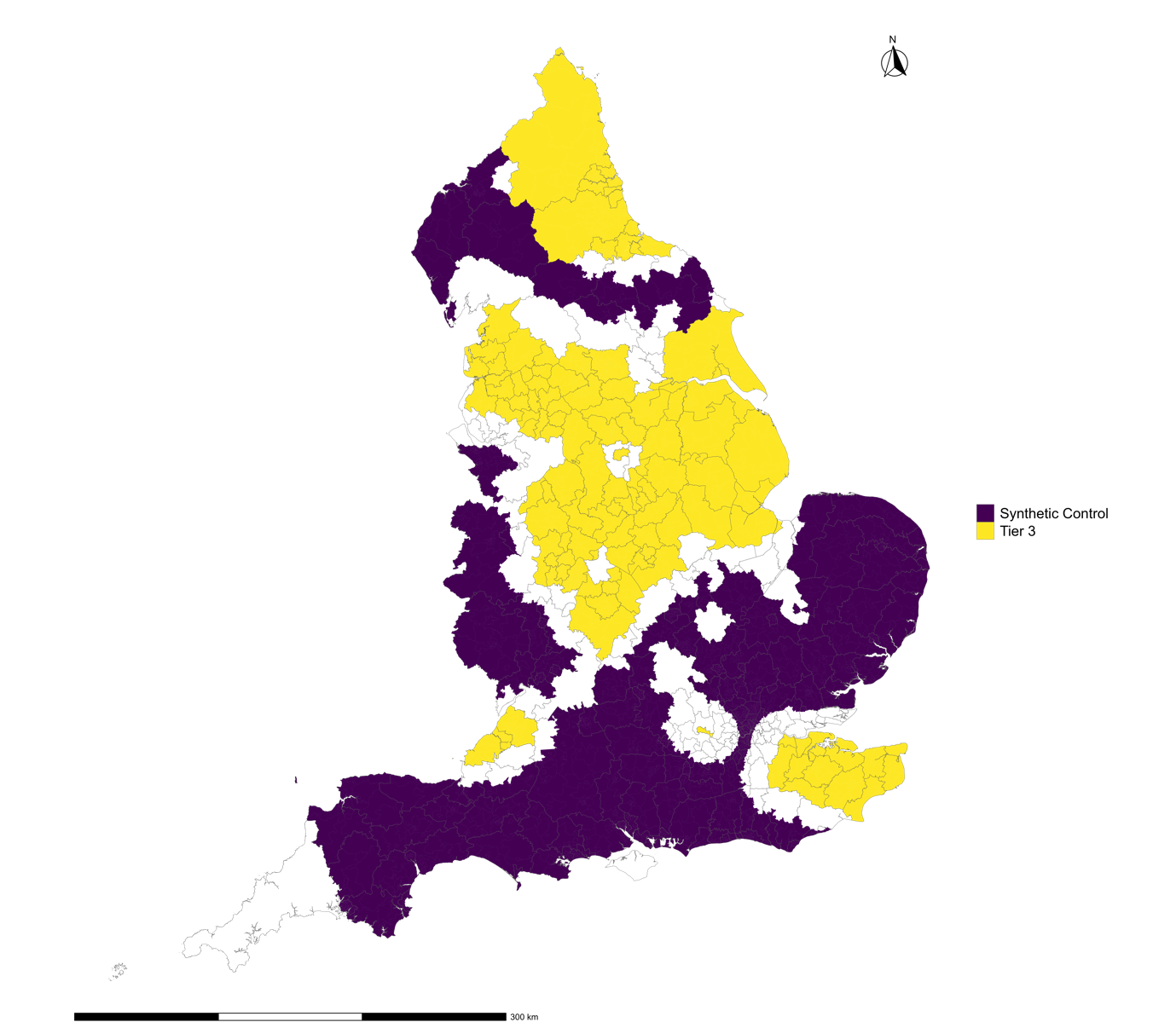


**Supplement 5: Location of Tier 2 areas used to construct synthetic control group.**

**Figure SF4. Location of synthetic control (purple; Tier 2) and intervention (Tier 3) areas (yellow) by Local Authorities.**


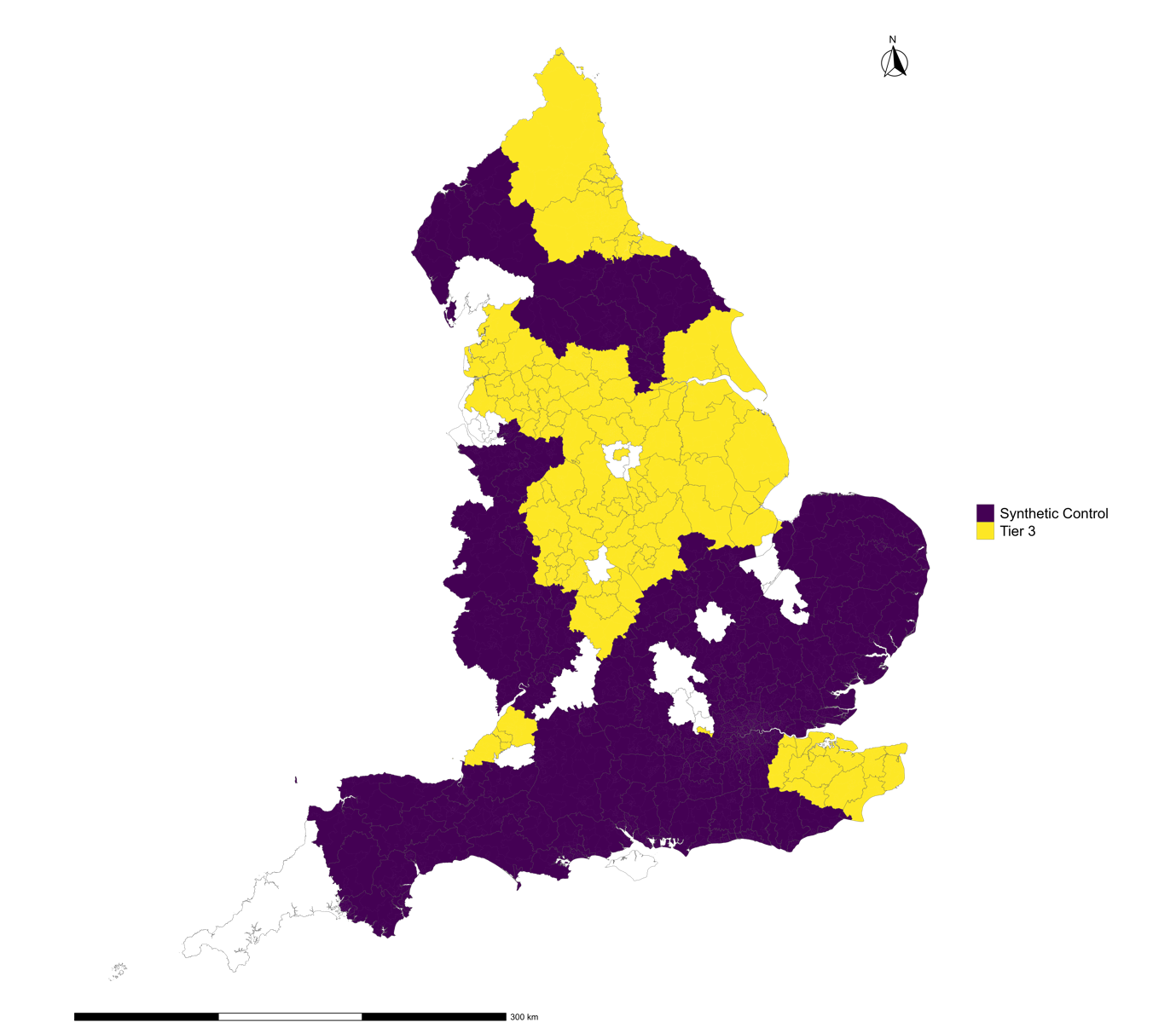

Supplement: online supplemental file 1 [file bmjopen-15-1-s001.docx]
