# Supplementary material for: Impact of tiered restrictions in December 2020 on COVID-19 hospitalisations in England: a synthetic control study
Source: BMJ Open. 2025 Jan 4;15(1):e086802. doi: 10.1136/bmjopen-2024-086802 (PMC11749879; doi:10.1136/bmjopen-2024-086802)
Supplement: Supplementary file 2 [file bmjopen-2024-086802.reviewer_comments.pdf]

PEER REVIEW HISTORY

BMJ Open publishes all reviews undertaken for accepted manuscripts. Reviewers are asked to complete a checklist review form and are provided with free text boxes to elaborate on their assessment. These free text comments are reproduced below.

ARTICLE DETAILS

Title (Provisional)

Impact of tiered restrictions in December 2020 on covid-19 hospitalisations in England: a synthetic control study.

Authors

Zhang, Xingna; Hungerford, Daniel; Green, Mark A; Garcia-Finana, Marta; Buchan, Iain Edward; Barr, Ben

---

VERSION 1 - REVIEW

**Reviewer**	**1**
**Name**	**House, Thomas**
**Affiliation**	**University of Manchester, School of Mathematics**
**Date**	**09-May-2024**

COI **I have some interactions with some authors as would be expected for scientists working in a similar area but I do not believe I have a vested interest in this specific study.**

This paper uses a synthetic control approach to assessment of the impact of Tier 3 vs Tier 2 restrictions on COVID hospitalisation. In this approach, the controls are weighted during a common period of time in Tier 2 then compared when intervention sites are moved to Tier 3. My view of this approach is that it is appropriate to the research question and provided it was performed as outlined this makes the paper technically sound, subject to the limitations that are honestly described

A couple of minor issues might be good to address in a revised version:

(1) As I recollect it the evidence that Alpha was not more severe is ambiguous, maybe reviewing evidence in each direction beyond reference 40 would be valuable.

(2) Related to this, infectious diseases have their own dynamics, meaning that the usual (in)dependence assumptions of dependent variables in regression do not always hold (i.e. hospitalisations would be expected to cluster due to infection leading to localised clusters linked by infection chains). This should be stated as a limitation - there is no current straightforward way that I am aware of to combine its solution (fitting a mechanistic model)

with the benefits of a synthetic control approach so it does not invalidate the study, but should be borne in mind. In fact this is somewhat addressed in the Supplement 4 analysis.

**Reviewer**	**2**
**Name**	**Coccia, Mario**
**Affiliation**	**Yale University School of Medicine**
**Date**	**19-May-2024**
**COI**	**NONE**

Evaluating the impacts of tiered restrictions introduced in England in December 2020 on covid-19 hospitalisations: a synthetic control study

The topics of this paper are interesting, though well known. The structure and content must be revised, and results have to be better explained by authors before to be reconsidered for publication.

Title has to be shorter.

Abstract has to clarify the goal, and health and social implications of targeted restrictions. Moreover the spatial aspects is important, but also the duration has to be clarified.

Introduction has to better clarify the research questions of this study and provide more theoretical background. Authors have to better describe the different sources of transmission dynamics of COVID-19, e.g., trade, climate etc.…role of non-pharmaceutical measures, length of lockdown on COVID-transmission and socioeconomic systems, etc.…After that authors can focus on the topics of this study to provide a correct analysis for fruitful discussion. (See suggested readings that must be all read and used in the text).

Methods of this study is not clear. The section of Materials and methods must be re-structured with following three sections and same order:

• Research setting, sample and data

• Measures of variables

• Data analysis procedure.

Authors have to avoid a lot of subheadings that create fragmentation and confusion. If necessary, can use bullet points (same comments for section of results and all sections).

Results.

Table 1 does not consider a lot of factor…avoid acronyms such as MSOA, write full words…to be clear…

Figure 2 should also consider the different variants of COVID-19 because some variants such as DELTA they have more transmission, and same restrictions can produce different effects. Authors have to consider all these aspects….

Authors do not consider also other factors such as Pelzman effects, change of behaviour of people, etc.…

Analysis is partial, in any case all these factors have to be discussed, localized restrictions is only one factors over time people change behaviour...

To reiterate, avoiding a lot of sub-headings that create fragmentation of the paper.

Discussion.

First, authors have to synthesize the main results in a simple table to be clear for readers and then show what this study adds compared to other studies. As said all factors mentioned before have to be discusses, variants, Pelzman effects, etc.….Strengths and limitations can be better represented in a SWOT matrix to be more effective healthcare management for future pandemics.

Conclusion has not to be a poor summary, but authors have to focus on manifold limitations of this study and provide implications of health and social policy, as well as how nations can prevent next pandemics with vaccination and nonpharmaceutical measures of control.

Overall, then, the paper is interesting, but contribution is not clear and non-consistent, considering the manifold factors. Theoretical framework is weak, and some results create confusion… structure of the paper has to be improved; study design, discussion and presentation of results have to be clarified using suggested comments.

I strongly suggest improving the paper, by using all comments (suggested papers included to read and use all) that I will in-depth verify, and maybe it can be considered. If the paper is not improved as suggested it will be dismissed.

Suggested readings of relevant papers that have to be read and all inserted in the text and references.

Moniz, M., Soares, P., Nunes, B. et al. Is a tiered restrictions system an effective intervention for COVID-19 control? Results from Portugal, November-December 2020. BMC Public Health 24, 956 (2024). https://doi.org/10.1186/s12889-024-18369-1

Coccia M. 2021. The relation between length of lockdown, numbers of infected people and deaths of COVID-19, and economic growth of countries: Lessons learned to cope with future pandemics similar to COVID-19. Science of The Total Environment, vol. 775, n. 145801, https://doi.org/10.1016/j.scitotenv.2021.145801

Davies, N. G., Barnard, R. C., Jarvis, C. I., Russell, T. W., Semple, M. G., Jit, M., Edmunds, W. J., Centre for Mathematical Modelling of Infectious Diseases COVID-19 Working Group, & ISARIC4C investigators (2021). Association of tiered restrictions and a second lockdown with

COVID-19 deaths and hospital admissions in England: a modelling study. The Lancet. Infectious diseases, 21(4), 482–492. https://doi.org/10.1016/S1473-3099(20)30984-1

Askitas, N., Tatsiramos, K., Verheyden, B. 2021. Estimating worldwide effects of non-pharmaceutical interventions on COVID-19 incidence and population mobility patterns using a multiple-event study (Open Access)(2021) Scientific Reports, 11 (1), art. no. 1972.

Benati I.,Coccia M. 2022. Effective Contact Tracing System Minimizes COVID-19 Related Infections and Deaths: Policy Lessons to Reduce the Impact of Future Pandemic Diseases. Journal of Public Administration and Governance, vol. 12, n. 3, pp. 19-33. DOI: https://doi.org/10.5296/jpag.v12i3.19834

Li, Y., Undurraga, E. A., & Zubizarreta, J. R. (2022). Effectiveness of Localized Lockdowns in the COVID-19 Pandemic. American journal of epidemiology, 191(5), 812–824. https://doi.org/10.1093/aje/kwac008

Coccia M. 2023. Sources, diffusion and prediction in COVID-19 pandemic: lessons learned to face next health emergency[J]. AIMS Public Health, 2023, 10(1): 145-168. doi: 10.3934/publichealth.2023012

Murphy, C., Lim, W. W., Mills, C., Wong, J. Y., Chen, D., Xie, Y., Li, M., Gould, S., Xin, H., Cheung, J. K., Bhatt, S., Cowling, B. J., & Donnelly, C. A. (2023). Effectiveness of social distancing measures and lockdowns for reducing transmission of COVID-19 in non-healthcare, community-based settings. Philosophical transactions. Series A, Mathematical, physical, and engineering sciences, 381(2257), 20230132. https://doi.org/10.1098/rsta.2023.0132

Coccia M. 2022. Preparedness of countries to face COVID-19 pandemic crisis: Strategic positioning and underlying structural factors to support strategies of prevention of pandemic threats, Environmental Research, Volume 203, n. 111678, https://doi.org/10.1016/j.envres.2021.111678.

Núñez-Delgado A., Bontempi E., Coccia M., Kumar M., Farkas K., Domingo, J. L. 2021. SARS-CoV-2 and other pathogenic microorganisms in the environment, Environmental Research, Volume 201, n. 111606, https://doi.org/10.1016/j.envres.2021.111606.

Pavani, J., Cerda, J., Gutiérrez, L., Varas, I., Gutiérrez, I., Jofré, L., Ortiz, O., & Arriagada, G. (2022). Factors associated to the duration of COVID-19 lockdowns in Chile. Scientific reports, 12(1), 9516. https://doi.org/10.1038/s41598-022-13743-8

Coccia M. 2021. The impact of first and second wave of the COVID-19 pandemic: comparative analysis to support control measures to cope with negative effects of future infectious diseases in society. Environmental Research, vol. 197, June, n. 111099, https://doi.org/10.1016/j.envres.2021.111099

Caristia, S., Ferranti, M., Skrami, E., Raffetti, E., Pierannunzio, D., Palladino, R., Carle, F., Saracci, R., Badaloni, C., Barone-Adesi, F., Belleudi, V., Ancona, C., & AIE working group on

the evaluation of the effectiveness of lockdowns (2020). Effect of national and local lockdowns on the control of COVID-19 pandemic: a rapid review. Epidemiologia e prevenzione, 44(5-6 Suppl 2), 60–68. https://doi.org/10.19191/EP20.5-6.S2.104

Bontempi E., Coccia M., Vergalli S., Zanoletti A. 2021. Can commercial trade represent the main indicator of the COVID-19 diffusion due to human-to-human interactions? A comparative analysis between Italy, France, and Spain, Environmental Research, vol. 201, Article number 111529, https://doi.org/10.1016/j.envres.2021.111529

Karatayev, V. A., Anand, M., & Bauch, C. T. (2020). Local lockdowns outperform global lockdown on the far side of the COVID-19 epidemic curve. Proceedings of the National Academy of Sciences of the United States of America, 117(39), 24575–24580. https://doi.org/10.1073/pnas.2014385117

Coccia M. 2022. COVID-19 pandemic over 2020 (with lockdowns) and 2021 (with vaccinations): similar effects for seasonality and environmental factors. Environmental Research, Volume 208, 15 May 2022, n. 112711. https://doi.org/10.1016/j.envres.2022.112711

Flaxman, S., Mishra, S., Gandy, A., Unwin, H.J.T., Mellan, T.A., Coupland, H., Whittaker, C., (...), Bhatt, S. 2020. Estimating the effects of non-pharmaceutical interventions on COVID-19 in Europe (Open Access), (2020) Nature, 584 (7820), pp. 257-261.

Coccia M. 2021. Pandemic Prevention: Lessons from COVID-19. Encyclopedia, vol. 1, n. 2, pp. 433-444. doi: 10.3390/encyclopedia1020036

Coccia M. 2023. Effects of strict containment policies on COVID-19 pandemic crisis: lessons to cope with next pandemic impacts. Environmental science and pollution research international, 30(1), 2020–2028. https://doi.org/10.1007/s11356-022-22024-w

Coccia M. 2022. COVID-19 Vaccination is not a Sufficient Public Policy to face Crisis Management of next Pandemic Threats. Public Organization Review, https://doi.org/10.1007/s11115-022-00661-6

Manica, M., Guzzetta, G., Riccardo, F. et al. Impact of tiered restrictions on human activities and the epidemiology of the second wave of COVID-19 in Italy. Nat Commun 12, 4570 (2021). https://doi.org/10.1038/s41467-021-24832-z

Coccia M., Benati I. 2024. Negative effects of high public debt on health systems facing pandemic crisis: Lessons from COVID-19 in Europe to prepare for future emergencies[J]. AIMS Public Health, 2024, 11(2): 477-498. doi: 10.3934/publichealth.2024024

Smith, L. E., Potts, H. W. W., Amlôt, R., Fear, N. T., Michie, S., & Rubin, G. J. (2022). Tiered restrictions for COVID-19 in England: knowledge, motivation and self-reported behaviour. Public health, 204, 33–39. https://doi.org/10.1016/j.puhe.2021.12.016

VERSION 1 - AUTHOR RESPONSE

	Comment/ Recommendation details	Responses
**Reviewer: 1**<br>**Dr. Thomas House, University of Manchester**	This paper uses a synthetic control approa[illegible] restrictions on COVID hospitalisation. In th[illegible] period of time in Tier 2 then compared wh[illegible] approach is that it is appropriate to the res[illegible] outlined this makes the paper technically s[illegible] described.	Thank you for your thorough review and positive feedback on our use of the synthetic control approach. We appreciate your acknowledgment.
	A couple of minor issues might be good to address in a revised version:<br>(1) As I recollect it the evidence that Alpha was not more severe is ambiguous, maybe reviewing evidence in each direction beyond reference 40 would be valuable.	Thank you for your valuable feedback. We agree that the severity of the Alpha variant has some ambiguity in the evidence. We have now broadened the scope of our literature review to include studies that present evidence both for and against increased severity, extending beyond the original reference 40. We have changed our text in lines 13-15 on page 14 to reflect this. Below are the newly added references for your information:<br><br>48. Davies NG, Jarvis CI, Edmunds WJ, Jewell NP, Diaz-Ordaz K, Keogh RH. Increased mortality in community-tested cases of SARS-CoV-2 lineage B.1.1.7. Nature. 2021;593(7858):270-274. doi:10.1038/s41586-021-03426-1<br><br>49. Goethem NV, Vandromme M, Oyen HV, et al. Severity of infection with the SARS-CoV-2 B.1.1.7 lineage among hospitalized COVID-19 patients in Belgium. PLOS ONE. 2022;17(6):e0269138. doi:10.1371/journal.pone.0269138<br><br>50. Pascall DJ, Vink E, Blacow R, et al. The SARS-CoV-2 Alpha variant was associated with increased clinical severity of COVID-19 in Scotland: A genomics-based retrospective cohort analysis. PLOS ONE. 2023;18(4):e0284187. doi:10.1371/journal.pone.0284187
	(2) Related to this, infectious diseases have their own dynamics, meaning that the usual (in)dependence assumptions of dependent variables in regression do not always hold (i.e. hospitalisations would be expected to cluster due to	Thank you for raising this. We have now included it as a limitation in the discussion in lines 7-11 on page 15.

	infection leading to localised clusters linked by infection chains). This should be stated as a limitation - there is no current straightforward way that I am aware of to combine its solution (fitting a mechanistic model) with the benefits of a synthetic control approach so it does not invalidate the study, but should be borne in mind. In fact this is somewhat addressed in the Supplement 4 analysis.	
**Reviewer: 2 Dr. Mario Coccia, Yale University School of Medicine**	The topics of this paper are interesting, though well known. The structure and content must be revised, and results have to be better explained by authors before to be reconsidered for publication.	Thank you for your feedback. We appreciate your recognition of the relevance of the topics covered in the paper. We have now revised the manuscript to improve the structure and clarity of the content, and to better explain the results in a more cohesive and detailed manner.
	Title has to be shorter.	Done.
	Abstract has to clarify the goal, and health and social implications of targeted restrictions. Moreover the spatial aspects is important, but also the duration has to be clarified.	We have revised the Abstract accordingly in lines 2-8, 12-14, 16 and 18-19 on page 2.
	Introduction has to better clarify the research questions of this study and provide more theoretical background. Authors have to better describe the different sources of transmission dynamics of COVID-19, e.g., trade, climate etc.…role of non-pharmaceutical measures, length of lockdown on COVID-transmission and socioeconomic systems, etc.…After that authors can focus on the topics of this study to provide a correct analysis for fruitful discussion. (See suggested readings that must be all read and used in the text).	Thank you for your feedback. We appreciate your input on strengthening the theoretical background and clarifying the research questions. However, it seems there may be some misunderstanding regarding the focus of our study. Our research is centred on **hospitalisation as an indicator of healthcare burden** rather than on the transmission dynamics of COVID-19 itself. Consequently, topics such as trade, climate, and transmission dynamics are not directly relevant to our study's objectives.<br><br>We agree that the role of non-pharmaceutical interventions is essential in our analysis. As such, we have already dedicated a significant portion of the introduction to discussing their impact on hospitalisation rates, which aligns directly with our focus on healthcare outcomes.<br><br>While we appreciate your suggested readings, we believe that those related specifically to COVID-19 transmission dynamics fall outside the scope of our paper. Nevertheless, we have reviewed the recommendations and included

		those relevant at various places in the introduction. We are also conscious of the limited word count available and wanted to make sure we had enough space elsewhere for the other revisions.
	Methods of this study is not clear. The section of Materials and methods must be re-structured with following three sections and same order:<br>• Research setting, sample and data<br>• Measures of variables<br>• Data analysis procedure.<br>Authors have to avoid a lot of subheadings that create fragmentation and confusion. If necessary, can use bullet points (same comments for section of results and all sections).	Thank you for your feedback on the structure of the Methods section. However, we believe there may have been some misunderstanding regarding our original layout. We have now changed it to enhance clarity. Our current structure is designed to align with the journal's requirements and to logically convey our study's focus. Specifically:<br><br>1. **Patient and public involvement** is included at the beginning of the section as it is a mandatory requirement from the journal.<br><br>2. **Setting, data and measures** corresponds to the "Research setting, sample, and data" category you suggested. This section includes measure and variables. The original heading is "Data and setting". We have now changed it to address your suggestions. This change is highlighted in line 14 on page 6.<br><br>3. **Intervention** is a critical component of our study, and we have provided a dedicated sub-section to give it the necessary space and clarity. Given the complexity of our intervention, this detailed explanation is essential for proper understanding.<br><br>4. **Analysis** aligns directly with your suggested "Data analysis procedure" section. We have now merged the original sub-sections of "Analysis" and "Sensitivity analysis" here to incorporate your suggestions. These changes are highlighted in lines 9-10 on page 9.<br><br>We feel that the suggested structure, particularly the division between "Research setting, sample and data" and "Measures of variables," would introduce unnecessary overlap and disrupt the logical flow of our paper. Additionally, separating "Measures of variables"

		from "Data" could lead to confusion, as they are closely related in our study.<br><br>Regarding the use of subheadings, we have tried to merge some to avoid excessive fragmentation while still ensuring clarity. We believe that keeping the intervention as a distinct sub-section improves readability, given its central role in our research.
	Results.<br>Table 1 does not consider a lot of factor…avoid acronyms such as MSOA, write full words…to be clear…<br>Figure 2 should also consider the different variants of COVID-19 because some variants such as DELTA they have more transmission, and same restrictions can produce different effects. Authors have to consider all these aspects….<br>Authors do not consider also other factors such as Pelzman effects, change of behaviour of people, etc.…<br>Analysis is partial, in any case all these factors have to be discussed, localized restrictions is only one factors over time people change behaviour...<br><br>To reiterate, avoiding a lot of sub-headings that create fragmentation of the paper.	We have now included the discussion of unmeasured confounders in line 17 on page 14 in the limitations. In response to your suggestions on presentation, we have now clarified the acronyms "MSOA" and “LFT” in Table 1 on page 10-11 to improve readability.<br><br>We appreciate your concerns about accounting for additional covid-19 variants. We would like to clarify that our study period predates the emergence of the Delta variant (B.1.617.2) in England, which was first detected in late March 2021. Therefore, our analysis focuses on the Alpha variant (B.1.1.7), which was the primary variant of concern in England during our study period. We have therefore only accounted for the Alpha variant’s impact within our analysis. Our study design is not set up to say much about the Delta variant so we feel it is better to not mention it to avoid any confusion. We have now added texts in lines 9-10 on page 6 to highlight this.<br><br>We acknowledge that our synthetic control design may not fully capture underlying behavioural changes independent of the intervention. While we assume that behavioural trends in intervention and control areas would be comparable, given their similar observed characteristics and pre-COVID trends, it remains possible that some coincidental changes in behaviour may have occurred exclusively within the intervention areas, unrelated to the tiered restrictions. These coincidental changes could contribute to the observed effects and thus may influence the estimated effectiveness of the intervention. We have now included this limitation in the revised manuscript in lines 26-32 on page 14 and lines 1-6 on page 15, highlighting the potential impact of unobserved,

		coincidental behavioural changes on our findings.<br><br>Our results provide no evidence for considering Pelzman effects where safety interventions can lead to compensatory risk-taking behaviours, potentially reducing or counteracting the intended benefits of the intervention. We have also included the discussion of this in lines 3-6 on page 15 in the limitations.<br><br>To summarise, in responding to your suggestions to present our analysis in a more comprehensive way, we have added discussion of limitations of our analysis on various aspects mentioned above, including unmeasured confounders and behavioural changes at various places. To incorporate your suggestions to improve the flow and streamline the narrative, we have now merged the original sub-sections of “Statistical analysis” and “Sensitivity analysis” in lines 2-3 on page 13.
	Discussion.<br>First, authors have to synthesize the main results in a simple table to be clear for readers and then show what this study adds compared to other studies. As said all factors mentioned before have to be discusses, variants, Pelzman effects, etc.....Strengths and limitations can be better represented in a SWOT matrix to be more effective healthcare management for future pandemics.	We would like to clarify that, in this paper, our focus is on evaluating the effectiveness of localised interventions to reduce COVID-19 hospitalisations, rather than on providing specific healthcare management strategies for future pandemics. Consequently, a SWOT analysis, typically used for broader strategic assessments of healthcare resource management, is beyond the scope and intent of our study. Following the standard practice in similar studies, we therefore chose to discuss the strengths and limitations narratively, rather than in the format of a SWOT matrix in this section.<br><br>In terms of presenting results as tables in the Discussion, it is rather uncommon to include them in this section. We have instead synthesised our findings narratively to maintain alignment with standard practice in similar studies. Additionally, we have already considered the main circulating COVID-19 variant (Alpha) relevant to our study period (see lines 9-10 on page 6). Our results lend no support for the Pelzman effects in our paper (see lines 3-6 on page 15).
	Conclusion has not to be a poor summary, but authors have to focus on manifold limitations of this study and	We would like to clarify that our study primarily focuses on evaluating the effectiveness of nonpharmaceutical measures, specifically

	provide implications of health and social policy, as well as how nations can prevent next pandemics with vaccination and nonpharmaceutical measures of control.	localised restrictions, in reducing COVID-19 hospitalisations. In line with this focus, we have concentrated on pandemic preparedness through nonpharmaceutical interventions in the Conclusion. Vaccination strategies, while crucial in pandemic response, fall outside the scope of our study (our study period begins before the use of vaccination) and were therefore not discussed in depth. We believe this focus provides a more accurate reflection of our study's aims and findings.<br><br>We have detailed the limitations of interpretating our results in Discussion already. We followed the paper structure used in the journal and one preferred by places like STROBE (i.e., to keep limitations in the discussion). However, we have reviewed presentation of our conclusion and expanded on the implications of health and social policy for future crises in lines 26-31 on page 15, to ensure our conclusions are as comprehensive and impactful as possible.
	Overall, then, the paper is interesting, but contribution is not clear and non-consistent, considering the manifold factors. Theoretical framework is weak, and some results create confusion… structure of the paper has to be improved; study design, discussion and presentation of results have to be clarified using suggested comments.<br><br>I strongly suggest improving the paper, by using all comments (suggested papers included to read and use all) that I will in-depth verify, and maybe it can be considered. If the paper is not improved as suggested it will be dismissed.	Thank you for your detailed review of our manuscript and for the feedback provided. While we appreciate your comments, we believe there may have been some misunderstandings regarding key aspects of our study, including the study period, methodological approach, and research focus.<br><br>Our study specifically addresses the impact of localised restrictions during a pre-Delta period, accounting for the prevailing variant and behavioural changes at the time. We utilised a synthetic control method precisely to isolate the effects of these interventions, a distinction that seems to have been overlooked. We have, however, reviewed our presentation to further clarify these elements and strengthen the coherence of our theoretical framework and contribution at various places throughout the manuscript.<br><br>Regarding your request for additional references, we found it unusual to be asked to include 23 references, especially considering that several of these were already cited in our original manuscript. Among the remaining 20 suggested references, many of which are not directly relevant to our article's specific focus on quantifying causal effects of localised

		nonpharmaceutical interventions. Nevertheless, we have reviewed the recommendations and included those relevant at various places in Introduction.
	Suggested readings of relevant papers that have to be read and all inserted in the text and references.<br><br>Moniz, M., Soares, P., Nunes, B. et al. Is a tiered restrictions system an effective intervention for COVID-19 control? Results from Portugal, November-December 2020. BMC Public Health 24, 956 (2024). https://doi.org/10.1186/s12889-024-18369-1<br><br>Coccia M. 2021. The relation between length of lockdown, numbers of infected people and deaths of COVID-19, and economic growth of countries: Lessons learned to cope with future pandemics similar to COVID-19. Science of The Total Environment, vol. 775, n. 145801, https://doi.org/10.1016/j.scitotenv.2021.145801<br><br>Davies, N. G., Barnard, R. C., Jarvis, C. I., Russell, T. W., Semple, M. G., Jit, M., Edmunds, W. J., Centre for Mathematical Modelling of Infectious Diseases COVID-19 Working Group, & ISARIC4C investigators (2021). Association of tiered restrictions and a second lockdown with COVID-19 deaths and hospital admissions in England: a modelling study. The Lancet. Infectious diseases, 21(4), 482–492. https://doi.org/10.1016/S1473-3099(20)30984-1<br><br>Askitas, N., Tatsiramos, K., Verheyden, B. 2021. Estimating worldwide effects of non-pharmaceutical interventions on COVID-19 incidence and population mobility patterns using a multiple-event study (Open Access)(2021) Scientific Reports, 11 (1), art. no. 1972.<br><br>Benati I.,Coccia M. 2022. Effective	We already referenced the following articles in our original manuscript:<br><br>Flaxman, S., Mishra, S., Gandy, A., Unwin, H.J.T., Mellan, T.A., Coupland, H., Whittaker, C., (...), Bhatt, S. 2020. Estimating the effects of non-pharmaceutical interventions on COVID-19 in Europe (Open Access), (2020) Nature, 584 (7820), pp. 257-261.<br><br>Li, Y., Undurraga, E. A., & Zubizarreta, J. R. (2022). Effectiveness of Localized Lockdowns in the COVID-19 Pandemic. American journal of epidemiology, 191(5), 812–824. https://doi.org/10.1093/aje/kwac008.<br><br>Davies, N. G., Barnard, R. C., Jarvis, C. I., Russell, T. W., Semple, M. G., Jit, M., Edmunds, W. J., Centre for Mathematical Modelling of Infectious Diseases COVID-19 Working Group, & ISARIC4C investigators (2021). Association of tiered restrictions and a second lockdown with COVID-19 deaths and hospital admissions in England: a modelling study. The Lancet. Infectious diseases, 21(4), 482–492. https://doi.org/10.1016/S1473-3099(20)30984-1.<br><br>Three relevant references on quantifying effects of localised restrictions for COVID-19 that we included in lines 6-15 on page 5:<br><br>Manica, M., Guzzetta, G., Riccardo, F. et al. Impact of tiered restrictions on human activities and the epidemiology of the second wave of COVID-19 in Italy. Nat Commun 12, 4570 (2021). https://doi.org/10.1038/s41467-021-24832-z<br><br>Moniz, M., Soares, P., Nunes, B. et al. Is a tiered restrictions system an effective intervention for COVID-19 control? Results

	Contact Tracing System Minimizes COVID-19 Related Infections and Deaths: Policy Lessons to Reduce the Impact of Future Pandemic Diseases. Journal of Public Administration and Governance, vol. 12, n. 3, pp. 19-33. DOI: https://doi.org/10.5296/jpag.v12i3.19834<br><br>Li, Y., Undurraga, E. A., & Zubizarreta, J. R. (2022). Effectiveness of Localized Lockdowns in the COVID-19 Pandemic. American journal of epidemiology, 191(5), 812–824. https://doi.org/10.1093/aje/kwac008<br><br>Coccia M. 2023. Sources, diffusion and prediction in COVID-19 pandemic: lessons learned to face next health emergency[J]. AIMS Public Health, 2023, 10(1): 145-168. doi: 10.3934/publichealth.2023012<br><br>Murphy, C., Lim, W. W., Mills, C., Wong, J. Y., Chen, D., Xie, Y., Li, M., Gould, S., Xin, H., Cheung, J. K., Bhatt, S., Cowling, B. J., & Donnelly, C. A. (2023). Effectiveness of social distancing measures and lockdowns for reducing transmission of COVID-19 in non-healthcare, community-based settings. Philosophical transactions. Series A, Mathematical, physical, and engineering sciences, 381(2257), 20230132. https://doi.org/10.1098/rsta.2023.0132<br><br>Coccia M. 2022. Preparedness of countries to face COVID-19 pandemic crisis: Strategic positioning and underlying structural factors to support strategies of prevention of pandemic threats, Environmental Research, Volume 203, n.111678, https://doi.org/10.1016/j.envres.2021.111678.<br><br>Núñez-Delgado A., Bontempi E., Coccia M., Kumar M., Farkas K., Domingo, J. L. 2021. SARS-CoV-2 and other pathogenic	from Portugal, November-December 2020. BMC Public Health 24, 956 (2024). https://doi.org/10.1186/s12889-024-18369-1<br><br>Karatayev, V. A., Anand, M., & Bauch, C. T. (2020). Local lockdowns outperform global lockdown on the far side of the COVID-19 epidemic curve. Proceedings of the National Academy of Sciences of the United States of America, 117(39), 24575–24580. https://doi.org/10.1073/pnas.2014385117<br><br>One relevant reference on evaluating NPI effects focuses on viral transmission dynamics on national and global scales that we included in line 25 on page 4:<br><br>Askitas, N., Tatsiramos, K., Verheyden, B. 2021. Estimating worldwide effects of non-pharmaceutical interventions on COVID-19 incidence and population mobility patterns using a multiple-event study (Open Access)(2021) Scientific Reports, 11 (1), art. no. 1972.<br><br>Two relevant references on reviewing evidence of effects of national and localised restrictions for COVID-19 that we included in lines 27-29 on page 5:<br><br>Murphy, C., Lim, W. W., Mills, C., Wong, J. Y., Chen, D., Xie, Y., Li, M., Gould, S., Xin, H., Cheung, J. K., Bhatt, S., Cowling, B. J., & Donnelly, C. A. (2023). Effectiveness of social distancing measures and lockdowns for reducing transmission of COVID-19 in non-healthcare, community-based settings. Philosophical transactions. Series A, Mathematical, physical, and engineering sciences, 381(2257), 20230132. https://doi.org/10.1098/rsta.2023.0132<br><br>Caristia, S., Ferranti, M., Skrami, E., Raffetti, E., Pierannunzio, D., Palladino, R., Carle, F., Saracci, R., Badaloni, C., Barone-Adesi, F., Belleudi, V., Ancona, C., & AIE working group on the evaluation of the effectiveness of lockdowns (2020). Effect of national and local lockdowns on the

	microorganisms in the environment, Environmental Research, Volume 201, n. 111606, https://doi.org/10.1016/j.envres.2021.111606.<br><br>Pavani, J., Cerda, J., Gutiérrez, L., Varas, I., Gutiérrez, I., Jofré, L., Ortiz, O., & Arriagada, G. (2022). Factors associated to the duration of COVID-19 lockdowns in Chile. Scientific reports, 12(1), 9516. https://doi.org/10.1038/s41598-022-13743-8<br><br>Coccia M. 2021. The impact of first and second wave of the COVID-19 pandemic: comparative analysis to support control measures to cope with negative effects of future infectious diseases in society. Environmental Research, vol. 197, June, n. 111099, https://doi.org/10.1016/j.envres.2021.111099<br><br>Caristia, S., Ferranti, M., Skrami, E., Raffetti, E., Pierannunzio, D., Palladino, R., Carle, F., Saracci, R., Badaloni, C., Barone-Adesi, F., Belleudi, V., Ancona, C., & AIE working group on the evaluation of the effectiveness of lockdowns (2020). Effect of national and local lockdowns on the control of COVID-19 pandemic: a rapid review. Epidemiologia e prevenzione, 44(5-6 Suppl 2), 60–68. https://doi.org/10.19191/EP20.5-6.S2.104<br><br>Bontempi E., Coccia M., Vergalli S., Zanoletti A. 2021. Can commercial trade represent the main indicator of the COVID-19 diffusion due to human-to-human interactions? A comparative analysis between Italy, France, and Spain, Environmental Research, vol. 201, Article number 111529, https://doi.org/10.1016/j.envres.2021.111529<br><br>Karatayev, V. A., Anand, M., & Bauch, C. T. (2020). Local lockdowns outperform global lockdown on the far side of the	control of COVID-19 pandemic: a rapid review. Epidemiologia e prevenzione, 44(5-6 Suppl 2), 60–68. https://doi.org/10.19191/EP20.5-6.S2.104<br><br>For the rest of the references, we have decided not to include. The list of references below are those examined effects of COVID-19 control measures mainly from the aspects of infections, transmissions, death, or in the context of global or national lockdowns, or comparison of different countries or waves:<br><br>Benati I.,Coccia M. 2022. Effective Contact Tracing System Minimizes COVID-19 Related Infections and Deaths: Policy Lessons to Reduce the Impact of Future Pandemic Diseases. Journal of Public Administration and Governance, vol. 12, n. 3, pp. 19-33. DOI: https://doi.org/10.5296/jpag.v12i3.19834<br><br>Coccia M. 2021. The relation between length of lockdown, numbers of infected people and deaths of COVID-19, and economic growth of countries: Lessons learned to cope with future pandemics similar to COVID-19. Science of The Total Environment, vol. 775, n. 145801, https://doi.org/10.1016/j.scitotenv.2021.145801<br><br>Coccia M. 2023. Sources, diffusion and prediction in COVID-19 pandemic: lessons learned to face next health emergency[J]. AIMS Public Health, 2023, 10(1): 145-168. doi: 10.3934/publichealth.2023012<br><br>Coccia M. 2021. The impact of first and second wave of the COVID-19 pandemic: comparative analysis to support control measures to cope with negative effects of future infectious diseases in society. Environmental Research, vol. 197, June, n. 111099, https://doi.org/10.1016/j.envres.2021.111099<br><br>Bontempi E., Coccia M., Vergalli S., Zanoletti A. 2021. Can commercial trade represent the main indicator of the COVID-19 diffusion due to human-to-human

	COVID-19 epidemic curve. Proceedings of the National Academy of Sciences of the United States of America, 117(39), 24575–24580. https://doi.org/10.1073/pnas.2014385117<br><br>Coccia M. 2022. COVID-19 pandemic over 2020 (with lockdowns) and 2021 (with vaccinations): similar effects for seasonality and environmental factors. Environmental Research, Volume 208, 15 May 2022, n. 112711. https://doi.org/10.1016/j.envres.2022.112711<br><br>Flaxman, S., Mishra, S., Gandy, A., Unwin, H.J.T., Mellan, T.A., Coupland, H., Whittaker, C., (...), Bhatt, S. 2020. Estimating the effects of non-pharmaceutical interventions on COVID-19 in Europe (Open Access), (2020) Nature, 584 (7820), pp. 257-261.<br><br>Coccia M. 2021. Pandemic Prevention: Lessons from COVID-19. Encyclopedia, vol. 1, n. 2, pp. 433-444. doi: 10.3390/encyclopedia1020036<br><br>Coccia M. 2023. Effects of strict containment policies on COVID-19 pandemic crisis: lessons to cope with next pandemic impacts. Environmental science and pollution research international, 30(1), 2020–2028. https://doi.org/10.1007/s11356-022-22024-w<br><br>Coccia M. 2022. COVID-19 Vaccination is not a Sufficient Public Policy to face Crisis Management of next Pandemic Threats. Public Organization Review, https://doi.org/10.1007/s11115-022-00661-6<br><br>Manica, M., Guzzetta, G., Riccardo, F. et al. Impact of tiered restrictions on human activities and the epidemiology of the second wave of COVID-19 in Italy. Nat Commun 12, 4570 (2021). https://doi.org/10.1038/s41467-	interactions? A comparative analysis between Italy, France, and Spain, Environmental Research, vol. 201, Article number 111529, https://doi.org/10.1016/j.envres.2021.111529<br><br>Coccia M. 2022. COVID-19 pandemic over 2020 (with lockdowns) and 2021 (with vaccinations): similar effects for seasonality and environmental factors. Environmental Research, Volume 208, 15 May 2022, n. 112711. https://doi.org/10.1016/j.envres.2022.112711<br><br>Coccia M. 2023. Effects of strict containment policies on COVID-19 pandemic crisis: lessons to cope with next pandemic impacts. Environmental science and pollution research international, 30(1), 2020–2028. https://doi.org/10.1007/s11356-022-22024-w<br><br>The references below have topics outside the scope of our paper and thus are not included. They focused on lockdown durations, adherence to local restrictions, environmental factors, mobility patterns, vaccination, pandemic preparedness and prevention:<br><br>Pavani, J., Cerda, J., Gutiérrez, L., Varas, I., Gutiérrez, I., Jofré, L., Ortiz, O., & Arriagada, G. (2022). Factors associated to the duration of COVID-19 lockdowns in Chile. Scientific reports, 12(1), 9516. https://doi.org/10.1038/s41598-022-13743-8<br><br>Smith, L. E., Potts, H. W. W., Amlôt, R., Fear, N. T., Michie, S., & Rubin, G. J. (2022). Tiered restrictions for COVID-19 in England: knowledge, motivation and self-reported behaviour. Public health, 204, 33–39. https://doi.org/10.1016/j.puhe.2021.12.016<br><br>Núñez-Delgado A., Bontempi E., Coccia M., Kumar M., Farkas K., Domingo, J. L. 2021. SARS-CoV-2 and other pathogenic

	021-24832-z<br><br>Coccia M., Benati I. 2024. Negative effects of high public debt on health systems facing pandemic crisis: Lessons from COVID-19 in Europe to prepare for future emergencies[J]. AIMS Public Health, 2024, 11(2): 477-498. doi: 10.3934/publichealth.2024024<br><br>Smith, L. E., Potts, H. W. W., Amlôt, R., Fear, N. T., Michie, S., & Rubin, G. J. (2022). Tiered restrictions for COVID-19 in England: knowledge, motivation and self-reported behaviour. Public health, 204, 33–39. https://doi.org/10.1016/j.puhe.2021.12.016	microorganisms in the environment, Environmental Research, Volume 201, n. 111606, https://doi.org/10.1016/j.envres.2021.111606.<br><br>Coccia M. 2022. Preparedness of countries to face COVID-19 pandemic crisis: Strategic positioning and underlying structural factors to support strategies of prevention of pandemic threats, Environmental Research, Volume 203, n.111678, https://doi.org/10.1016/j.envres.2021.111678.<br><br>Coccia M. 2021. Pandemic Prevention: Lessons from COVID-19. Encyclopedia, vol. 1, n. 2, pp. 433-444. doi: 10.3390/encyclopedia1020036<br><br>Coccia M. 2022. COVID-19 Vaccination is not a Sufficient Public Policy to face Crisis Management of next Pandemic Threats. Public Organization Review, https://doi.org/10.1007/s11115-022-00661-6<br><br>Coccia M., Benati I. 2024. Negative effects of high public debt on health systems facing pandemic crisis: Lessons from COVID-19 in Europe to prepare for future emergencies[J]. AIMS Public Health, 2024, 11(2): 477-498. doi: 10.3934/publichealth.2024024

VERSION 2 - REVIEW

**Reviewer**	**1**
**Name**	**House, Thomas**
**Affiliation**	**University of Manchester, School of Mathematics**
**Date**	**16-Nov-2024**
**COI**	

No further comments.
